# Trajectories of Alcohol Use During the Transition to Adulthood

**Published:** 2004

**Authors:** Jennifer L. Maggs, John E. Schulenberg

**Affiliations:** Jennifer L. Maggs, Ph.D., is an associate professor of human development and family studies at the Pennsylvania State University, University Park, Pennsylvania. John E. Schulenberg, Ph.D., is a professor of psychology at the University of Michigan and research professor at the Institute for Social Research, both positions in Ann Arbor, Michigan

**Keywords:** young adult, adolescent, alcohol abuse, AOD (alcohol and other drug) consumption, AOD use pattern, AOD use initiation, age differences, risk factors, human study, longitudinal study, cross-sectional study, latent growth curve modeling, causal path analysis, multivariate analysis, multilevel analysis, normative trajectory, multiple-trajectory, prevention of AOD associated consequences

## Abstract

People’s alcohol use and abuse tend to increase, peak, and then decrease as they go through the transition to adulthood, a period that spans the late teenage years through the mid- to late twenties. However, more specific pathways, or trajectories, of alcohol use are embedded within the normative alcohol use pathway. Studying these trajectories of alcohol use can elucidate the origins and consequences of alcohol problems as well as guide prevention and treatment programs. Models of the average trend (i.e., normative trajectory approaches) are simpler than models that posit multiple trajectories and may replicate more consistently across samples and age spans. However, multiple-trajectory approaches allow for a more specific understanding of the origins, developmental course, and outcomes of alcohol use and abuse among adolescents and young adults.

This article considers the average, or normative, developmental course of alcohol use and abuse, as well as some common developmental pathways, or trajectories, embedded within the normative course. The article also reviews key research on the events and circumstances during adolescence that predict different paths of alcohol use, as well as outcomes during young adulthood that are associated with different trajectories.

In the years after people graduate from high school, they undergo major transitions in every domain of their lives. Young people may embark on diverse life paths (Schulenberg and Maggs 2002; Shanahan 2000), and the age and order in which they reach developmental milestones tend to vary widely (Cohen et al. 2003). Flexibility and self-direction in day-to-day life increase for many, and their geographic mobility may be greater than at any other time of life (Shanahan 2000). In the past few decades, this transition period has lengthened considerably, and people tend to begin to settle into adult roles, such as work and marriage, later than their counterparts in past generations. These changes have led Arnett (2000) to argue that this period should be viewed as unique and important in its own right rather than as simply a staging ground for adulthood.

On average, people start drinking during their adolescence, increase the amounts they drink into their early twenties, and decrease the amounts when they take on adult roles (Bachman et al. 2002; Johnston et al. 2004). Some personal and role changes (such as becoming a college student) coincide with these increases, and others (such as becoming a spouse, parent, or worker) coincide with the decreases (Schulenberg et al. 2003).

Many young people establish lifelong patterns of alcohol use (and nonuse) during this period of emerging adulthood. Others take a different trajectory, engaging in a particular pattern of use only in their late teen or young adult years and not thereafter. Variations in the timing and intensity of changes in alcohol consumption can be predicted partly on the basis of demographic, psychological, social, and behavioral factors. Hidden within the normative pattern are subgroups of people whose drinking patterns take different developmental trajectories over time. By identifying common trajectories of alcohol use and abuse across adolescence and young adulthood, researchers can better understand the origins of alcohol use disorders, predict different outcomes, and plan prevention and intervention programs. At a clinical level, knowledge of the drinking patterns that define the different subgroups can expedite the early identification of alcohol problems, facilitate diagnosis, and inform treatment (Flory et al. 2004; Sher et al. in press; Zucker 1995).

## Alcohol Use Patterns From Late Adolescence to Young Adulthood

Studies that aim to identify the causes and likely outcomes of young adults’ alcohol use typically consider the age at which young people begin drinking and the frequency and intensity of alcohol use (Maggs and Schulenberg 2005). One common approach to examining associations between drinking and age is to compare different age groups at once (cross-sectional studies). However, this method provides limited information about how a person’s alcohol use may change over time. Although cross-sectional studies can identify average developmental trends in alcohol use, they are less effective at predicting one person’s future drinking patterns based on his or her past and present alcohol use.

In contrast to cross-sectional studies, studies that track the same people over time (long-term longitudinal studies) allow investigators to examine why some people do not follow trajectories that seemed likely based on their earlier behavior (Schulenberg and Maggs 2002). Trajectory approaches using longitudinal data focus on a person’s course of alcohol use over several years.^1^ Some emphasize the trajectory that is most commonly observed within a population (normative trajectory approaches). A second kind of trajectory approach, known as a multiple-trajectory (or taxonomy) approach, focuses on distinct subgroups of people who follow similar trajectories. For example, a study using a multiple-trajectory approach might compare a group of people who are chronic heavy drinkers and a group of people who are decreasing their alcohol use. These approaches are discussed in the following sections.

### Normative Trajectory Approaches: Describing the Average Course

Many pivotal research questions pertain to the normative course of alcohol use and abuse as well as to how important factors associated with alcohol use, such as having friends who drink, are correlated and predict changes in drinking. Although not all people follow the same pathway, understanding the average or normative developmental trend is of significant value for science and for prevention.

One benefit of normative trajectory approaches is their parsimony: Statistical analyses that describe the average trend (and note individual differences around it) are simpler to understand than multiple-trajectory analyses because they focus on the total sample and tend to be less mathematically complex. Furthermore, because they focus on the total sample (rather than sample-specific subgroups, which might show greater variability across different studies), these models seem to replicate more consistently across samples and age spans. Finally, because they are less complex, normative trajectory approaches can more readily incorporate measures of stable factors (e.g., gender) and time-varying factors (e.g., having substance-using peers) that are associated with alcohol use patterns.

Two analytic methods used in normative trajectory approaches are latent growth curve (LGC) models and multilevel models (MLM). These statistical techniques mathematically summarize the behavior of a group of people across multiple occasions, yielding a trajectory, or curve. LGCs are well suited to examining whether trajectories of multiple variables are correlated. For example, Duncan and colleagues (1996) demonstrated that some adolescents’ substance use (first variable) trajectories were predicted by their older siblings’ substance use (second variable) trajectories across a 3-year period.

In addition to modeling cumulative developmental trajectories, MLMs also can be used to examine whether, over time, levels of a variable (e.g., alcohol use) rise and fall in tandem with other fluctuating variables (e.g., mood, time spent hanging out in unsupervised settings). For example, Maggs and Schulenberg (1998) showed that adolescents reported greater alcohol use on occasions when they gave more reasons in favor of drinking and fewer reasons against it, independent of the developmental trend toward more alcohol use.

### Taxonomy Approaches: Identifying Multiple Trajectories

An alternative research approach is to identify distinct, homogeneous subgroups of people whose alcohol use trajectories during the transition to adulthood differ from one another. These different trajectories may have different antecedents and consequences and may therefore require different theoretical explanations and approaches to prevention and health promotion.

In the related domain of antisocial behavior, Moffitt (1993) proposed a dual taxonomy distinguishing between adolescence-limited and life-course-persistent antisocial behavior. Both types of antisocial behavior involve more delinquent and criminal behavior in adolescence, but the former group only engages in antisocial behavior during adolescence, whereas the latter, much smaller life-course-persistent group becomes involved in antisocial behavior earlier and continues it longer. It is hypothesized that the causes, meaning, and consequences of the behaviors differ markedly between the groups.

Taxonomies also have been proposed for types of adult alcoholism. For example, early alcohol research distinguished early-onset, antisocial alcoholism from later-onset, primary alcoholism. Zucker (1995) proposed a more developmental approach, taking into account the antecedents, course, and outcomes of alcohol problems. He identified four subgroups: Antisocial Alcoholism, Developmentally Cumulative Alcoholism, Developmentally Limited Alcoholism (time-limited, peer-focused heavy drinking and spontaneous reduction with the successful assumption of family and career roles), and Negative Affect Alcoholism (the use of alcohol to modulate negative mood, characterized by later onset).

Longitudinal studies that track people’s alcohol use across multiple occasions from adolescence to adulthood also have identified taxonomies of young people with different trajectories of heavy alcohol use^2^ (see the figure). Some studies have spanned a broad age range, from early adolescence into the twenties (e.g., Chassin et al. 2002). Others have focused on subsets of this time period, either examining the subjects’ adolescent years up to age 18 (e.g., Hill et al. 2000) or beginning in the subjects’ late adolescence (e.g., prior to or just after high school) and following them into or through their twenties (e.g., Schulenberg et al. 1996; Sher et al. in press; Muthén and Muthén 2000). Studies also differ in the indicators of alcohol use considered (e.g., frequency of drinking, quantity consumed, frequency of heavy drinking,^3^ or alcohol-related consequences) and the types of samples included (e.g., nationally representative school-based and household samples, longitudinal followups of samples used in prevention studies, or high-risk samples). Despite these important methodological differences, and some differences in findings, consistent patterns have been identified.

**Figure:**
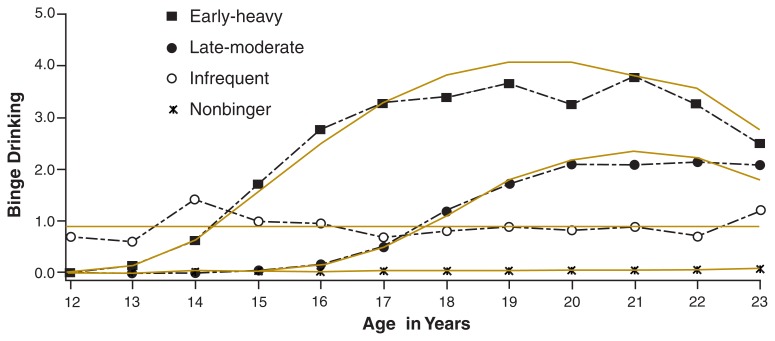
Trajectories of binge drinking from adolescence through emerging adulthood. Estimated growth trajectories for the three groups are indicated by solid gold lines. Dashed black lines represent observed means of binge drinking at each age for each group. Observed frequencies of binge drinking (past year) ranged from 0 (none) to 5 (1–2 times a week). NOTE: Early–heavy group, *N* = 99, 20.9 percent of the sample. Late–moderate group, *N* =134, 90.0 percent of the sample. Infrequent group, *N* = 43, 9.6 percent of the sample. Nonbinger group, *N* = 176, 30.5 percent of sample. SOURCE: Chassin et al. 2002. Copyright @2002 by the American Psychological Association. Reprinted with permission.

### Common Alcohol Use Trajectories

In research involving adolescents and young adults, the most common trajectory subgroup observed across studies contains abstainers, light drinkers, or very rare heavy drinkers across all time periods measured. Depending on definitions for these levels of alcohol consumption used in different studies, estimates of the proportion of young people in this low-risk group range from about one-fifth (e.g., White et al. 2000) to over two-thirds (e.g., Hill et al. 2000). Members of another common trajectory subgroup, stable-moderate drinkers, engage in some heavy drinking across adolescence and young adulthood but do not escalate or decelerate their use dramatically. Across studies, estimates are that about one-third of adolescents and emerging adults into the mid-twenties fall into this group (e.g., Chassin et al. 2002; Tucker et al. 2003). Together, these two broad categories—which comprise relatively low-risk drinkers—include a large proportion of all young people.

Many studies involving adolescents and young adults also have identified groups of chronic heavy drinkers and late-onset heavy drinkers. These groups are distinguished by the age when the subjects started heavy drinking, but as this age varies from study to study, chronic heavy drinkers and late-onset heavy drinkers are more difficult to identify or compare across studies. Those who are designated as chronic heavy drinkers typically start heavy drinking at younger ages, by middle adolescence (early onset), and tend not to decrease their drinking in their twenties (e.g., Bates and Labouvie 1997; Schulenberg et al. 1996; White et al. 2000). Members of the late-onset heavy-drinking subgroup start to drink later (i.e., middle to late high school) than stable-moderate and chronic heavy drinkers, but their use escalates steeply (e.g., Casswell et al. 2002; Flory et al. 2004; Muthén and Muthén 2000; Schulenberg et al. 1996; Tucker et al. 2003).

“Fling” drinkers (see Schulenberg et al. 1996), who make up 10 percent to 12 percent of the adolescent and young adult population, take yet a different trajectory. They experience a period of developmentally limited heavy drinking that peaks and then declines following late adolescence or the early adult years (e.g., Schulenberg et al. 1996; Tucker et al. 2003).

A final subgroup, decreasers, appears to be more common in older adolescent and young adulthood samples (e.g., Schulenberg et al. 1996) than in younger samples. Decreasers begin heavy drinking at an early age, such as in middle school, but reduce their consumption significantly during high school (Colder et al. 2002; Tucker et al. 2003). About 10 percent of adolescents and young adults fall into this subgroup.

Although researchers are beginning to understand what causes different people to follow these different trajectories, the applied and clinical significance of these pathways, while intriguing, has received little attention (Schulenberg and Maggs 2002). For example, universal school-based prevention programs that target the majority of early adolescents not engaging in any heavy drinking may be less successful with adolescents who are on early-onset trajectories.

### Advantages and Disadvantages of Trajectory Approaches

Normative trajectory and multiple-trajectory approaches, both of which emphasize assessing the subjects’ alcohol and other drug use at multiple times, have many advantages (as well as some disadvantages, primarily increased methodological and logistical complexity) compared with approaches that measure the subjects’ drinking behavior only on one or two occasions. Both types of trajectory approaches, compared with one- and even two-time-point approaches, offer the potential for far greater understanding of the causes, developmental course, and consequences of the subjects’ substance use (Schulenberg et al. 2003). Identifying the course of the subjects’ alcohol and other drug use across multiple measurement periods makes it possible to consider how people’s substance use changes over time, together with other factors, and to better understand the risk factors for and consequences of different alcohol use patterns. This is especially important during adolescence and young adulthood, because people tend to experiment with alcohol and other drugs during this time, and substance use at a given time may have little relation to a young person’s later patterns of use or abuse.

One important disadvantage of trajectory approaches is shared with all longitudinal studies: Participants with different characteristics and group memberships may drop out of the study at different rates.

A noteworthy limitation of both normative and multiple-trajectory approaches, compared with other longitudinal approaches, is that trajectory approaches traditionally tend to downplay day-to-day situational factors that may affect people’s likelihood of drinking, such as negative mood or the peers a person is hanging out with on a specific day (Maggs and Schulenberg 1998). In addition, such approaches tend not to focus on short-term consequences of alcohol use. To better identify and understand short-term fluctuations in alcohol use, some studies repeatedly measure subjects’ behavior over short periods of time, for example using beepers or Web-based or paper diaries.

Importantly, methodological variations in different studies may contribute to different conclusions about alcohol use trajectories during the transition to adulthood. These include the operational definition of heavy drinking that is used, the characteristics of the sample such as age range or level of alcohol use, and the number of and interval between measurement occasions.

Normative and multiple-trajectory approaches each have distinct advantages and disadvantages, such that the advantage of one is the other’s disadvantage. Researchers should determine which type of trajectory approach to use by the research questions they are addressing and the data characteristics they are using. Because normative approaches tend to be more straightforward and parsimonious than multiple-trajectory approaches, it is better to use a normative trajectory approach when the only difference among multiple-trajectory groups is the *amount* of substance use. As researchers have learned, however, the *shapes* of people’s alcohol use trajectories often differ. This fact encourages the use of a multiple-trajectory approach that recognizes variability both across people and over time, rather than assuming homogeneity of the directions and rates of change within the entire population. This increased focus on identifying different developmental trajectories of alcohol use across adolescence and adulthood represents a major and welcome shift in researchers’ ability to understand the meaning and course of alcohol use and problems across a person’s life course. Advantages of the multiple-trajectory approach over the normative trajectory approach include being able to determine the specific risk and protective factors that apply to particular subgroups and being able to focus more on understanding the meaning of a person’s behaviors in the context of his or her developmental history.

The multiple-trajectory approach also has important limitations. First, there is a danger that subgroups, identified as sets of tendencies and probable characteristics, may come to be seen as immutable, almost as diagnostic categories. This may be premature, because application of these analytic strategies to alcohol use trajectories is relatively new, and further research is needed to determine which subgroups will be found consistently across different samples and measures of alcohol use. Furthermore, the number of trajectory subgroups identified is logically dependent on the sample characteristics; the number, range, and spacing of observations; and the way variables are operationalized and measured.

Before findings derived from multiple-trajectory approaches can be translated into diagnostic tools, researchers need to have greater understanding about whether different risk factors can reliably distinguish different subgroups, either before members of these subgroups diverge from the normative pattern or during their divergence. Likewise, it is important to remember that a person’s membership in a class or subgroup is probabilistic rather than absolute. Moreover, applied work using multiple-trajectory approaches to examine alcohol use patterns has tended not to emphasize variability among people within subgroups.

Finally, a noteworthy methodological limitation of multiple-trajectory approaches is that the range of ages included in a given study determines the study’s ability to distinguish some subgroups from others. For example, studies that end when the subjects reach age 20 may inaccurately categorize some people as late-onset heavy drinkers, when instead they subsequently may reduce their drinking and therefore would be better described as fling drinkers (e.g., Chassin et al. 2002; Flory et al. 2004; Hill et al. 2000). Similarly, because studies that begin in late adolescence lack data regarding alcohol use during early adolescence, they cannot distinguish decreasers from potential “early flings” (e.g., Hill et al. 2000; Schulenberg et al. 1996).

## Predictors and Correlates of Trajectory Membership

Numerous longitudinal studies have examined predictors (typically measured early in the study) and outcomes of belonging to particular trajectory subgroups, such as role status or problem behaviors at or after the final assessment period used to identify the subgroups. Some of these, and their significance, are described in the following sections.

### Predictors of Trajectory Subgroup Membership

Several studies show that whether adolescents or young adults should be designated as belonging in heavier-drinking subgroups can be predicted by whether or not they have several pre-existing personal and social–environmental characteristics: being male, not living with two biological parents, and having parents with heavier alcohol use and greater symptoms of alcoholism or antisocial personality (Casswell et al. 2002; Chassin et al. 2002; Hill et al. 2000; Schulenberg et al. 1996; White et al. 2000). During late adolescence and early adulthood, male heavy drinkers are especially likely to have already exhibited externalizing symptoms such as aggressive behaviors and early delinquent behavior (Chassin et al. 2002; Flory et al. 2004; Hill et al. 2000; Tucker et al. 2003).

Alcohol-related factors that predict whether young people will embark on subsequent heavier-drinking trajectories include the degree to which they have more positive expectancies about alcohol’s effects, whether they think it is all right to drink to get drunk or drink to cope with stressful situations, and whether they have prior alcohol and drug use (Chassin et al. 2002; Flory et al. 2004; Schulenberg et al. 1996).

Other risk factors include certain personal characteristics, involvement with social institutions, and peer influence. People who have such personal attributes as higher sensation-seeking and lower conventionality, self-esteem, and self-efficacy also are at risk for following heavier-drinking trajectories (Flory et al. 2004; Schulenberg et al. 1996; Tucker et al. 2003). Low involvement with social institutions also is associated with greater risk; for example, students with poorer performance in school, lack of commitment to attending classes, and less church involvement are more likely to be in heavy-drinking subgroups (Flory et al. 2004). Finally, peer factors are important predictors of riskier trajectories, with peer alcohol use, perceived peer approval of substance use, and less resistance to peer pressure each predicting heavier alcohol use (Flory et al. 2004; Chassin et al. 2002; Schulenberg et al. 1996; Tucker et al. 2003). These risk factors are similar to those observed in more traditional cross-sectional and longitudinal research (see Hawkins et al. 1992 for a review).

### Trajectory Outcomes for Young Adults

A series of recent studies has examined whether young adults’ health or the rate at which they attain various social roles is affected by their trajectories of alcohol use.

Research indicates that high school students who do not drink heavily are more likely to complete high school and attend college than their peers who do drink heavily (Chassin et al. 2002; Hill et al. 2000; Muthén and Muthén 2000; Tucker et al. 2003). However, many young people only begin binge drinking or drinking heavily during college (Schulenberg et al. 1996).

Criminal behavior, including stealing, selling drugs, and committing violent acts, is more prevalent among heavier drinkers in general (Hill et al. 2000; Tucker et al. 2003). Binge drinkers also are more likely to exhibit symptoms of antisocial personality disorder than people who do not binge drink (Chassin et al. 2002). However, compared with young people who rarely use alcohol, fling drinkers—whose heavy drinking spikes during late adolescence and young adulthood and then declines—tend to have equivalent psychosocial adjustment during adulthood (Schulenberg et al. 2003).

Health in young adulthood also differs by alcohol use trajectories. One study found that at age 24, people who had been classified as chronic heavy drinkers based on their trajectories of drinking in adolescence had increased risk for obesity, hypertension, and illness, compared with peers who had not been heavy drinkers during adolescence (Oesterle et al. 2004). These findings were maintained even when gender, ethnicity, poverty, and current level of alcohol and other substance use were statistically controlled (Oesterle et al. 2004). Chronic binge drinkers and late-onset drinkers also reported more sex partners by age 21 than nonbinge drinkers (Guo et al. 2002). People who were not binge drinkers had the most positive health behaviors and status, and increasers and late-onset binge drinkers generally fell between the chronic binge and nonbinge drinking groups in terms of this variable. Risk for alcohol and other drug abuse and dependence in young adulthood is lowest among people who do not engage in heavy episodic, or binge, drinking (Chassin et al. 2002; Hill et al. 2000; Muthén and Muthén 2000). Many studies show few or no health differences between light drinkers and abstainers, although some research suggests that health and personal relationships may be more positive among light drinkers than among abstainers.

## Conclusions and Implications

Several conclusions from these longitudinal studies on alcohol use trajectories deserve to be highlighted. First, many adolescents and young adults never engage in heavy drinking. Although the majority of people report some alcohol use, significant numbers never report drinking heavily at any assessment period. Although the proportion of young people who never drink heavily remains uncertain, studies indicate that it is somewhere between one-third (e.g., Colder et al. 2002) and more than two-thirds (Hill et al. 2000), depending on sample characteristics and the definition of heavy drinking used.

Second, the age when drinking (especially heavy drinking) begins is important. Meaningful differences in the predictors, course, and consequences of alcohol use can be found between the subgroup of people who begin drinking in early adolescence and those who begin drinking during middle or later adolescence (Flory et al. 2004; Maggs and Schulenberg 2005). Later onset of developmentally limited heavy drinking is more common than early onset, is associated with fewer early risk factors, and is determined more by situational and developmentally normative risk factors such as living with peers in college residences (Schulenberg and Maggs 2002). However, later onset of heavy drinking also carries health and other risks, as described previously (Chassin et al. 2002; Oesterle et al. 2004; Schulenberg and Maggs 2002).

Third, members of most subgroups reduce their alcohol use by their mid-twenties. Declines in use are associated with acquiring adult roles, such as spouse, parent, and worker (see the sidebar by O’Malley which follows this article).

Fourth, young people who consistently drink heavily, although few in number, have the most early and ongoing risk factors for behavioral and adjustment difficulties generally and the fewest positive adult outcomes (Muthén and Muthén 2000).

Fifth, early but infrequent binge drinkers may be at risk for late-onset alcohol problems associated with negative affect regulation (Chassin et al. 2002; Colder et al. 2002; Zucker 1995).

### Implications for Prevention and Intervention

What do the concepts of normative and multiple trajectories of alcohol use mean for prevention? The study of trajectory approaches has reinforced the ideas that (1) people develop in multiple dimensions across the adolescent and young adult years, (2) alcohol use behaviors change differently for different people, and (3) factors that predict alcohol use patterns also emerge and disappear at different ages. For these reasons, multiple dynamic approaches to prevention and intervention are needed. Clearly, one approach will not fit all needs. For example, early onset of infrequent use associated with negative affect may be rare, but the pattern deserves attention because young people who fit this trajectory are at risk for longer-term problems, including depression. Early onset of substance use predicts ongoing heavier use and associated difficulties for many, so middle-school drug use prevention programs are appropriate for this subgroup. Likewise, late onset of alcohol and other drug use with escalation also is harmful, so prevention and intervention efforts in high school and beyond also are needed. Recognizing the varied and dynamic trajectories that alcohol use can take, rather than viewing drinking as a static behavior, offers a stronger developmental foundation for effective interventions (Maggs and Schulenberg 1998; Schulenberg and Maggs 2002).

## Footnotes

1 A major distinction between trajectory models and other longitudinal analyses is that nontrajectory longitudinal analyses compare only two time points. Even if data are collected on multiple occasions, the analyses predict Time 2 from Time 1, and Time 3 from Time 2, and so on. In contrast, trajectory approaches map the cumulative progression of behavior over time.

2 Various analytic techniques are used to identify trajectory groups, ranging from cluster analysis to growth mixture modeling. Discussion of these techniques is beyond the scope of this article.

3 This article defines heavy drinking in general as consuming several drinks per occasion; often in the literature, this pattern of alcohol use is viewed more specifically as binge drinking, in which a person drinks five or more drinks on a single occasion.
